# VALIDATION AND CLINICAL USEFULNESS OF A SLEEP HEALTH SCALE IN LATE LIFE

**DOI:** 10.1093/geroni/igz038.1937

**Published:** 2019-11-08

**Authors:** Scott Ravyts, Joseph Dzierzewski, Elliottnell Perez, Emily Donovan

**Affiliations:** Virginia Commonwealth University, Richmond, Virginia, United States

## Abstract

Sleep health is a multidimensional construct of sleep and wakefulness which can be conceptualized as the opposite of sleep dysfunction. Assessing sleep health is particularly relevant among older adults who disproportionally experience sleep-related adverse outcomes. Yet, empirically-validated sleep health scales are lacking. The objectives of the present study were to assess the psychometric properties of a newly designed measure of sleep health (RU-SATED) among older adults and examine the association between sleep health and well-being in late-life. Data included 773 older adults (M=67.68, 52% female) who completed an online survey of their sleep and health. Respondents completed the six-item RU-SATED scale, the Insomnia Severity Index (ISI), and the Satisfaction with Life Scale (SWLS). Sleep health scores ranged from 1 to 12, (M=8.13, SD=2.68), with higher scores indicating better sleep health. Exploratory factor analysis revealed a one-factor model. Confirmatory factor analysis showed that a one-factor model was associated with model fit indices in the adequate range. Additionally, a hierarchical linear regression indicated that sleep health was positively associated with life satisfaction (β=.25, p<.001) and accounted for significant variance in life satisfaction above and beyond insomnia severity (∆R2=.04, p<.001). In conclusion, RU-SATED appears to be a valid measure of sleep health among older adults with potentially useful clinical applications. Future research would benefit from examining the association between sleep health and other relevant health outcomes, as well as assessing the prospective ability of sleep health to predict relevant outcomes above and beyond traditional measures of sleep quality or insomnia.

